# The South African Rugby Injury and Illness Surveillance and Prevention Project (SARIISPP): The Currie Cup Premiership Competition Injury Surveillance Report, 2023

**DOI:** 10.17159/2078-516X/2025/v37i1a21507

**Published:** 2025-04-15

**Figure:**
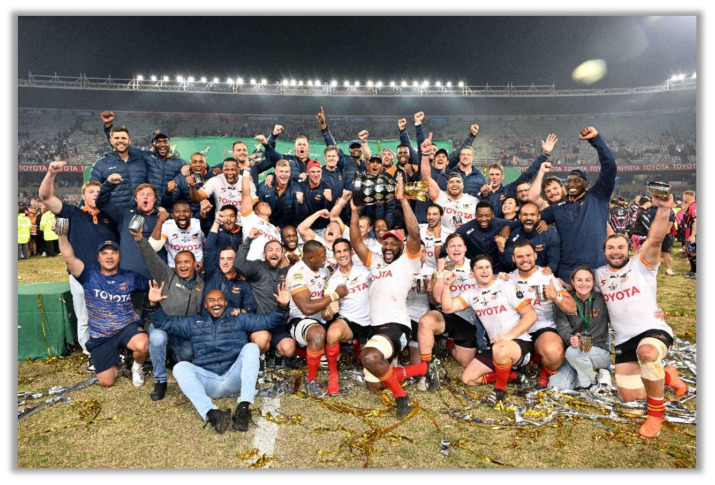


## Executive Summary

As part of the South African Rugby Injury and Illness Surveillance and Prevention Project (SARIISPP), the medical doctors and medical support staff of the respective teams recorded the injury data for the annual Currie Cup 2023 Premiership Division Competition (‘Currie Cup’). SARIISPP has been collecting and analysing these data annually since 2014 for the Currie Cup tournament. Historically, there have only been seven teams participating in this tournament. However, 2023 was the first year the Griffons joined the Currie Cup competition, bringing the total to eight teams. All eight teams must record the injuries that occur in each match and training session in their team throughout the season. The strength and conditioning coaches also recorded their training session data to calculate training exposure data throughout the season. By combining match exposure data, training exposure data, and injury data, SARIISPP aims to obtain a complete understanding of the factors contributing to player injury risk.

The analysis reveals injury patterns and facilitates comparison across different years, tournaments, teams, the scientific literature, and internationally. During this investigation, areas of concern are identified, and appropriate changes in the game, tournament structure, or medical support services are considered based on the evidence. Furthermore, when evidence supports such actions, injury-specific interventions can be developed and implemented.

This report uses injury burden and injury rate as key metrics for analysis. It is worth noting that even if teams maintain a low injury rate, high-severity injuries can still impose a substantial burden on the team. Such injuries lead to a significant number of training and match days lost due to injury. This highlights the importance of collecting data on injury severity instead of relying solely on injury rates.

The injury rates are expressed as the mean (95% confidence intervals) per 1000 player exposure hours. The injury rate of Time-Loss injuries for the Currie Cup 2023 was 64 (54 to 75) injuries per 1000 player hours; the lowest injury rate recorded since 2014. This is also significantly lower than the international meta-analysis injury rate of 91 (77 to 106) injuries per 1000 player hours at a comparative level of play [1]. Although the injury rate is significantly lower, it falls well within the expected limits of season-to-season variation for the Currie Cup. This equates to 1.3 injuries per team per match or roughly 4 injuries for every 3 matches played, with an average injury burden of 1232 days lost per 1000 player hours.

The Toyota Free State Cheetahs, who subsequently won the tournament, had the highest injury rate for Time-Loss injuries throughout the Currie Cup 2023 tournament. Windhoek Draught Griquas had a significantly lower injury rate in 2023 than their 2014–2022 tournament average. The Fidelity ADT Lions had the highest average severity of 32 days absent per injury, whereas the HOLLYWOODbets Sharks had the lowest average severity of 12 days absent per injury. The average severity of Time-Loss injuries in the 2023 tournament was 19 days, lower than the 27 days reported in the international meta-analysis [1]. The median injury severity of all Time-Loss injuries was 12 days, with 25% of injuries lasting 9 days or less and 25% lasting 24 days or more due to injury.

The most common injury type observed during the 2023 Currie Cup tournament was central nervous system injuries. Ligament sprain injuries, and muscle (rupture/strain/tear) injuries, ranked second and third, respectively.

The head, knee, and shoulder were the most injured body locations in that order. Shoulder injuries have decreased since the 2022 season. Head injuries increased by thirteen percent and knee injuries increased by seven percent. The number and incidence of concussions also increased in the 2023 Currie Cup tournament to an injury incidence of 18 (13 to 24) concussions per 1000 player hours: the highest recorded to date in this tournament. *Open play* at 20 (15 to 26) injuries per 1000 player hours, accounted for the most injuries in the 2023 Currie Cup tournament, followed by *Tackling* and *Being tackled* at 14 (10 to 19) injuries per 1000 player hours and 13 (9 to 18) injuries per 1000 player hours respectively.

A total of 61 Time-Loss training injuries were sustained in the Currie Cup 2023. During the tournament, 29% of Time-Loss injuries occurred during training. This equates to an incidence of 1.5 (1.1 to 1.8) injuries per 1000 training exposure hours, which is lower than the injury incidence of 3 (1.9 to 4.0) injuries per 1000 training hours reported in the meta-analysis [1]. Time-Loss training injuries resulted in an average severity of 17 days absent. *Semi-contact skills* accounted for the highest number of injuries during training for the 2023 Currie Cup tournament, with *Open play* being the largest injury-causing event.

**Figure:**
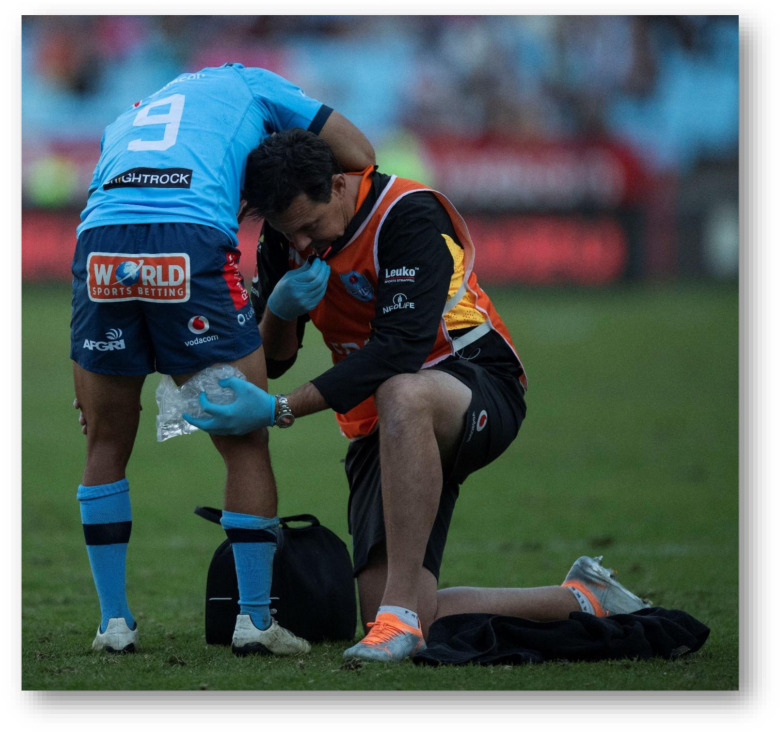


**Figure:**
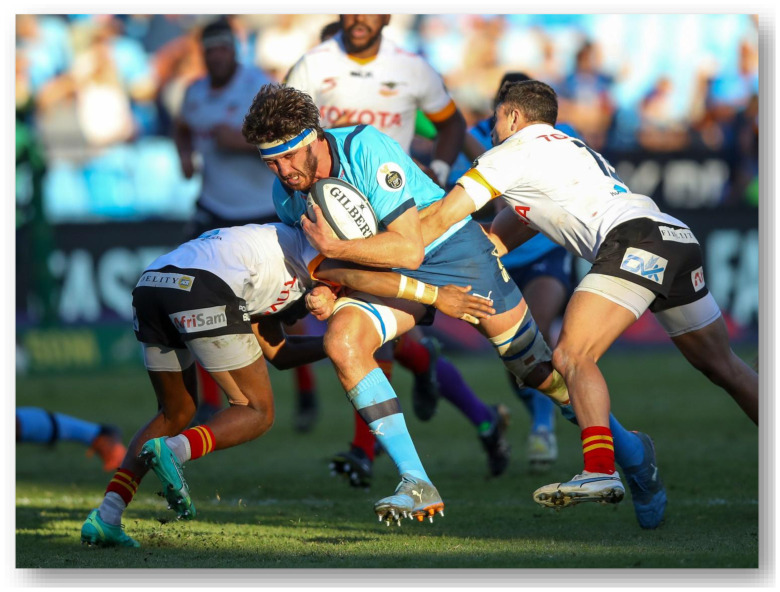


## Introduction

In 2014, the South African Rugby Union (SA Rugby) introduced a standardised injury surveillance format for the Currie Cup Premiership Division Competition as part of the South African Rugby Injury and Illness Surveillance and Prevention Project (SARIISPP). This format required the team’s medical doctor or medical support staff to record all relevant injury data from matches and training sessions using the standardised *BokSmart* injury surveillance data capture format. The definitions and reporting format used in this system are aligned with the IOC consensus statement for injury recording in sport [2], and with the consensus statement on injury definitions and data collection procedures for studies of injuries in rugby union [3].

Injury surveillance is an essential step in injury prevention. Specifically, it is important for developing injury prevention strategies and assessing their efficacy and effectiveness after implementation. By capturing injury surveillance data in a standardised format, it becomes possible to compare injury rates between teams participating in the same tournament, track tournament injuries over consecutive years, and compare findings with other rugby injury surveillance studies. This standardised approach enables comprehensive analysis and enhances the ability to make well-informed evidence-based decisions regarding injury patterns and potential prevention strategies.

Reports on rugby tournament injuries typically present the injury numbers as a rate (or incidence), i.e., the total number of injuries divided by the total amount of time exposed to the risk of experiencing an injury. The standardised format in this paper is to present the number of injuries per 1000 player exposure hours. Match exposure hours are calculated as the number of matches played multiplied by the number of exposed players (30) and the match duration (80 mins); for team-specific match-related exposure, 15 players would be utilised. Training exposure hours are calculated as the average number of players present at training multiplied by the average time spent training each week. These values are then summed to obtain the training exposure hours over the competition period. In this report, the standardised injury rates have been provided to allow for comparison with other reports. Every effort has been made to present these rates on a ‘per team’ and ‘per match’ basis for easier and more practical interpretation.

Since 2016, the Currie Cup medical doctors and medical support staff were asked to record the injured players’ physical return to play date, thereby calculating the actual severity of the injury. Injury burden is a combination of the injury rate and severity and is expressed as the number of days absent from training and matches per 1000 player hours. Throughout this report, only actual rather than predicted severity is used for analysis.

The report includes data from the 2014 and 2015 seasons only in sections reporting on injury numbers and incidence. The sections reporting on injury severity and burden begin with the 2016 season, the first time, actual severity data was collected.

In the Currie Cup 2020/21 seasonal report, the South African Rugby Injury and Illness Surveillance and Prevention Project (SARIISPP) began capturing Time-Loss training injuries and training exposure data. This addition enables SARIISPP to gain a more comprehensive understanding of injury data by combining match and training exposure and injury data.

An inherent bias with most injury surveillance studies is that the teams’ medical doctors or medical support staff are responsible for entering their team’s injury data. As no audit process is done on collecting these data, in many cases, the accuracy of the data depends on the compliance of the medical doctors or medical support staff. This potential limitation is present in most injury surveillance studies. SARIISPP had a project coordinator who communicated regularly with the medical doctors or support staff to minimise this potential limitation. This ensured that data capturing was up to date.

The Currie Cup 2023 semi-finals were contested between Airlink Pumas and HOLLYWOODbets Sharks and Vodacom Blue Bulls, and Toyota Free State Cheetahs. The final was between the Toyota Free State Cheetahs and Airlink Pumas, with the Toyota Free State Cheetahs eventually winning the tournament.

**Figure:**
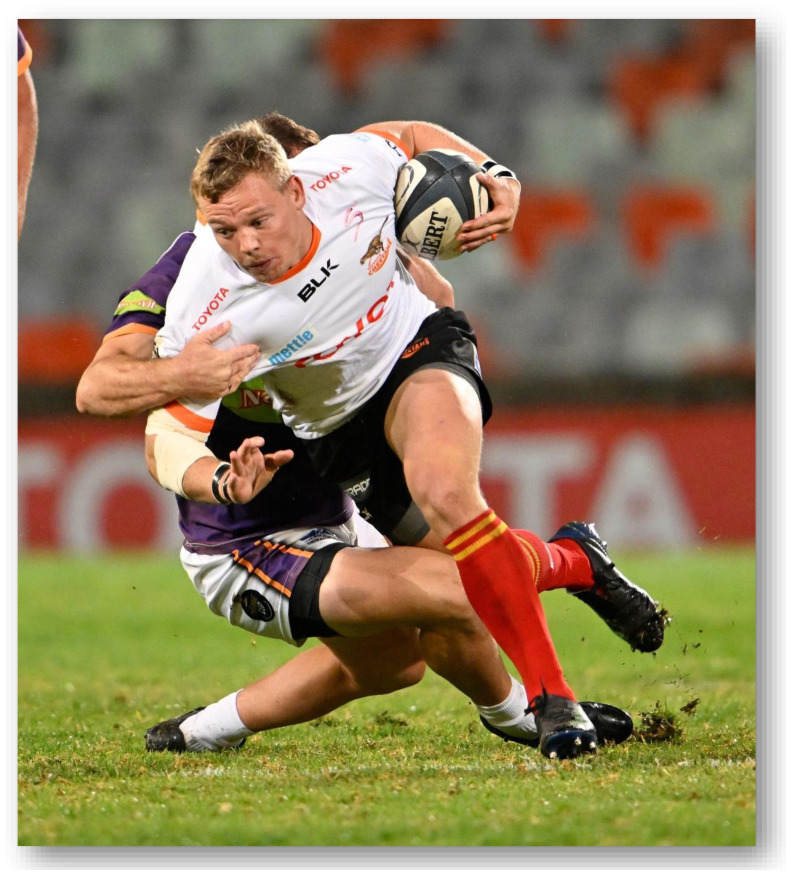


## Definitions

All definitions are originally based on the 2007 consensus statement for injury reporting in rugby union [3] and have since been realigned with the latest International Olympic Committee (IOC) consensus statement for methods of recording and reporting epidemiological data on injury and illness in sport [2].

### MEDICAL ATTENTION INJURY

All injuries seen by the teams’ medical doctors or medical support staff were classified as Medical Attention injuries. These injuries are defined by the 2007 statement as an “*injury that results in a player receiving medical attention”* [3], and by the more recent IOC statement as *“a health problem that results in an athlete receiving medical attention”* [2].

### TIME-LOSS INJURY

Medical Attention injuries were further categorised as Time-Loss injuries, where appropriate, and defined by the 2007 statement as “*an injury that results in a player being unable to take a full part in future rugby training or match play*” [3]. The IOC definition is *“a health problem that results in a player being unable to complete the current or future training session or competition”* [2]. For clarity, this means an injury sustained by a rugby union player during a match or training session that prevented or would have prevented the player from taking full part in all rugby training activities and/or match play for more than 1 day following the day of injury, irrespective of whether match or training sessions were scheduled [4].

### INJURY RATE

This report defines an injury rate as the number of injuries per 1000 player exposure hours. This method of expressing injury rate has been used in previous years’ reports of the Currie Cup Premiership tournament and other international literature, making comparisons easy. Moreover, the injury rate is expressed as a mean with 95% confidence intervals. A 95% confidence interval around a mean value indicates a 95% chance (i.e., very high chance) that the true value falls within this range. In this report, we present the 95% confidence intervals assuming a normal distribution of the data and use the approach of examining the overlap of the confidence intervals to determine whether the injury incidences are significantly different; if the range of confidence interval values of two comparisons does not overlap, there is a strong chance (95%) that their injury rates are different from each other. We have opted for this method because it is easy to use, conservative and less likely to produce false positive results [5].

### MEDIAN (INTERQUARTILE RANGE)

When numbers are ordered from the lowest to highest, the median separates the higher half of the values from the lower half. Simply put, it is the middle value of a list of ranked numbers. The interquartile range (IQR) describes the spread of the data. When rank-ordered data are divided into quartiles the first and the third quartiles represent the value under which 25% and 75% of the data points fall, respectively. For example, consider a team with a median injury severity of 32 days (IQR 7 to 40). This means that when the teams’ injury severities are ranked in order, the mid-point or median of the injury severities is 32 days. Also, 25% of their injuries result in 7 or fewer days absent from training and matches, and 25% result in 40 days or more absent from training and matches.

### NEW, SUBSEQUENT AND RECURRENT INJURIES

In 2023, in the Currie Cup Premiership Division Competition, a ‘*New Injury’* was defined as when a player sustained his first injury. Any injury the *same* player sustained after this initial injury was defined as a *‘Subsequent Injury’*.

According to the IOC statement, any subsequent injury to the same site and of the same type is referred to as a ‘*Recurrence’* if the index injury was fully recovered before reinjury and as an *‘Exacerbation’* if the index injury was not yet fully recovered [2].

To provide more detail on the subsequent injuries for practitioners, we have further categorized the subsequent injuries in this report into one of four groups based on the Orchard Sports Injury and Illness Classification System (OSIICS) classification diagnosis:

- Different site - Different type
- Different site - Same type
- Same site - Different type
- Same site - Same type

According to the 2007 Consensus Statement for rugby, any subsequent injury classified as ‘Same site - Same type’ was a *‘Recurrent injury’* [3].

### INJURY SEVERITY

The total severity of an injury is defined as *“the number of days that have elapsed from the date of injury to the date of the player’s return to full participation in team training and availability for match selection”* [2,3]. The actual severity of each injury is classified by the severity groupings provided in the 2007 consensus statement: *Slight* (0–1 days lost), *Minimal* (2–3 days lost), *Mild* (4–7 days lost), *Moderate* (8–28 days lost), *Severe* (>28 days lost), *Career ending*, and *Non-fatal catastrophic* [3]. To align with the latest IOC statement, the injuries have been re-grouped to reflect the severity groupings *‘1–7 days’, ‘8–28 days’ and ‘>28 days’* [*2*].

The average severity represents the average number of days lost per injury when dividing the accumulated total number of days lost by the total number of injury events. For example, a team may have a total severity of 550 days absent, accumulated from 22 injuries. The average severity of the team’s injuries would, therefore, be 550/22, which equals, on average, 25 days absent per injury.

### INJURY BURDEN

Injury burden is determined by the combination of injury rate and severity. It is calculated by multiplying the injury rate by the average severity (number of days lost due to injury). It is expressed as the number of days absent per 1000 player hours. For example, consider a team with an injury rate of 75 injuries per 1000 player exposure hours and an average severity of 38 days lost per injury. In this case, the injury burden for the team would be calculated as 2850 days absent per 1000 player hours (i.e., 75 × 38 = 2850).

### OPERATIONAL INJURY BURDEN

The operational burden is the expected number of days lost due to injury per team for every match played over the tournament or season. The measure extrapolates injury rates and severities over a season and includes the most severe and least severe injuries in its estimation. For example, suppose a team has an operational injury burden of 2 days. In that case, it means that based on their injury rates and average severity, and from within the team, 2 playing or training days lost due to injury can be expected from every match the team plays.

### META-ANALYSIS

A meta-analysis uses statistical methods to combine multiple scientific studies with varying levels of evidence on the same topic. The goal is to determine overall defining patterns and results based on the combined data. As such, it represents the highest level of scientific evidence available. The findings in this report are compared to the data in the most recent meta-analysis, which was published in 2021. The meta-analysis specifically focuses on rugby union injuries at an elite professional level [1].

**Figure:**
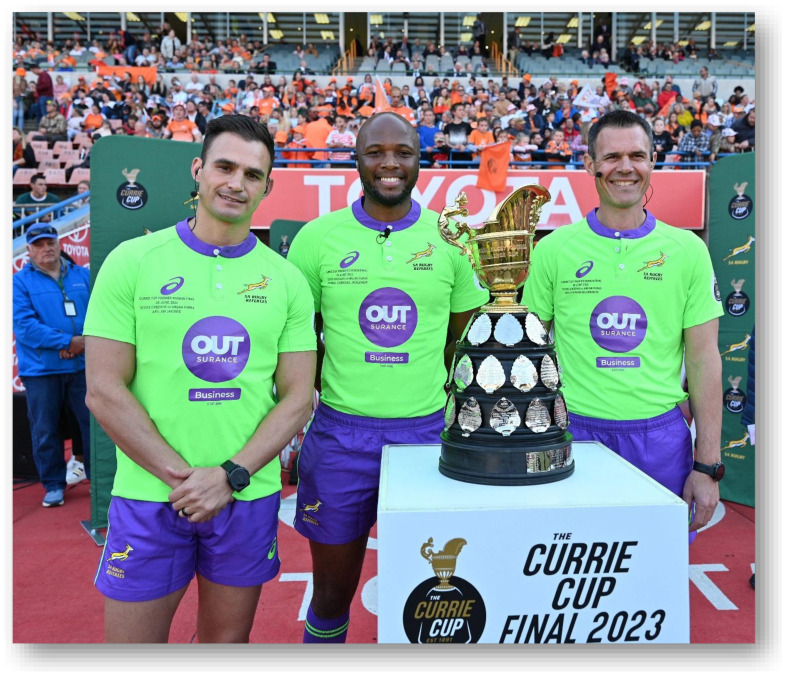


## MATCH INJURIES

### Injured players

During the Currie Cup 2023, 119 players sustained 152 match Time-Loss injuries. Due to squad changes over the tournament duration for various reasons, a total number of 316 different players were physically exposed to injury at some point while playing rugby matches as part of the Currie Cup 2023 tournament. However, for analysis and exposure calculation purposes, we assumed 184 players were available to play rugby on match days in the tournament (8 teams × 23 players per match-day squad). Sixty-five percent (65%) of the 184 available match-day players sustained a match injury during the tournament (Figure 1a). The proportion of players who sustained one and two Time-Loss injuries was the same in 2023 and 2022. Furthermore, the proportion of players who experienced 3 injuries increased slightly from 2022 to 2023 (Figure 1b). There were no players who sustained more than 3 injuries in 2023. Only the absolute number of Time-Loss injuries were analysed further in this report (n = 152), regardless of the number of players who sustained them.

### Overall Injury Rate

Only Time-Loss injuries have been analysed in this report because these injuries are more comparable between different teams, tournaments and with the published scientific literature [1].

**Figure 2:**
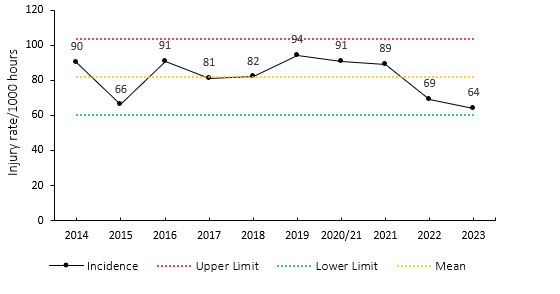
Injury incidence of Time-Loss match injuries over the surveillance period with mean ± standard deviations shown. The red dotted line represents the mean plus standard deviation. The green dotted line represents the mean minus standard deviation. 2020/21 – was a hybrid tournament structure that started in 2020 and carried over into the beginning of the 2021 season due to COVID-19 lockdown interruptions.

The overall match injury incidence for the Currie Cup 2023 was 64 (54 to 75) injuries per 1000 player exposure hours, the lowest injury rate in the last 10 years. The 2023 Currie Cup tournament’s injury rate is significantly different to the international meta-analysis injury rate of 91 (77 to 106) injuries per 1000 player hours [1] but falls within the season-to-season variation for the Currie Cup, based on the last 9 years’ collective data (Figure 2). An injury incidence of 64 injuries per 1000 player hours equates to 1.3 injuries per team per match or roughly 4 injuries for every 3 matches played.

**Figure 3:**
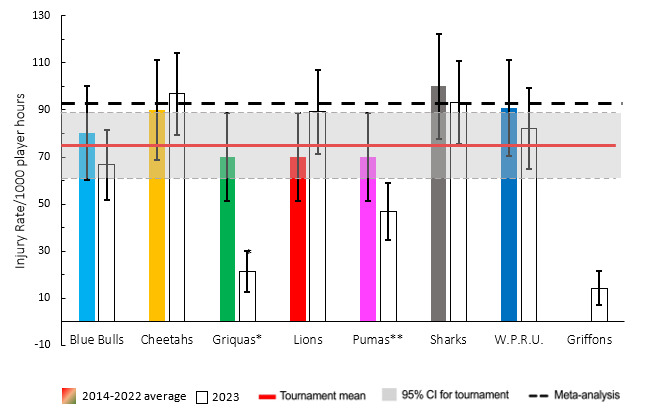
Injury incidence rates for Time-Loss injuries experienced by each team in the Currie Cup 2023 compared to their 2014–2022 averaged injury rate. (**) Average injury rates for Pumas 2015 – 2022 and Griquas for 2015, 2016, 2018, 2019, 2020/21, 2021 and 2022. Asterisk (*) indicates that a team’s 2023 injury rate significantly differs from its 2014–2022 average injury rate. The whisker lines for each bar represent the 95% Confidence Interval.

When comparing the team’s 2014–2022 averaged tournament injury incidence to their 2023 season’s injury incidence data, the Windhoek Draught Griquas experienced a significantly lower injury incidence rate in 2023 (Figure 3). The Airlink Pumas, in 2023, experienced an injury incidence rate lower than the grouped tournament average to date. No team showed significantly higher injury incidences than their 2014–2022 tournament averages.

Overall, the combined average injury incidence of 75 (61 to 89) injuries per 1000 player hours for all the teams over the last 10 years is similar to the international meta-analysis summary of 91 (77 to 106) injuries per 1000 player hours [1].

**Figure:**
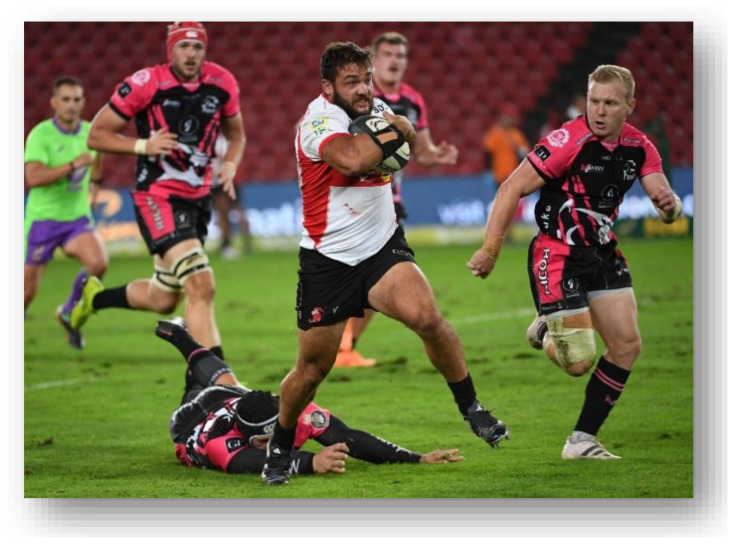


**Figure:**
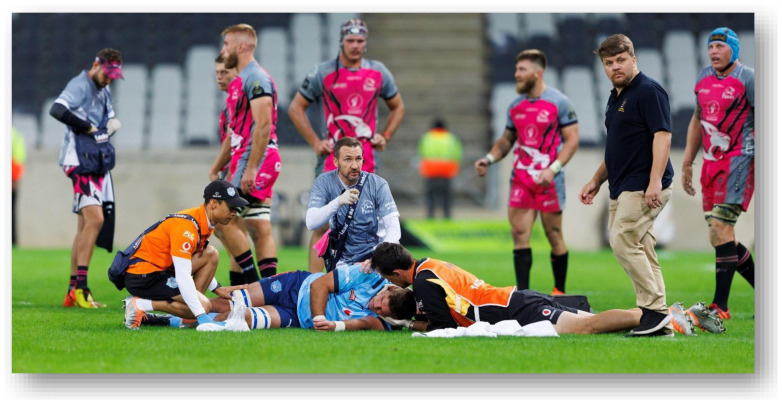


### Injury incidence over the season

**Figure 4:**
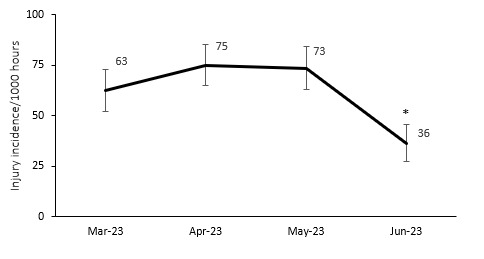
Match injury incidence per month of the 2023 Currie Cup season. Asterisk (*) indicates that the injury incidence is significantly lower in June than in March, April, May. The whiskers for each point represent the 95% Confidence Intervals.

The 2023 Currie Cup tournament consisted of two rounds of matches and took place from March to June. This year’s format was a double-round competition in the first half of the year, the same as in 2022. Table 1 shows the different tournament formats from 2014 to 2023. When examining the Time-Loss injury incidence during the 2023 Currie Cup tournament, it was found that the injury incidence in June was significantly lower than in March, April, and May. There were no significant differences between the other months in the 2023 season (Figure 4).

### Overall Severity

**Figure 5:**
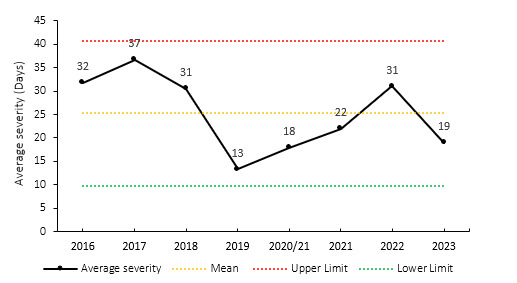
Mean severity of Time-Loss match injuries over the surveillance period with mean ± standard deviations shown. The red dotted line represents the mean plus standard deviation. The green dotted line represents the mean minus standard deviation. 2020/21 – was a hybrid tournament structure that started in 2020 and continued into the beginning of the 2021 season due to COVID-19 lockdown interruptions.

The average severity of match injuries for the Currie Cup 2023 was 19 days, which is within the expected season-to-season variation (Figure 5). Match injury severity increased consistently from 2019 to 2022 and then decreased in 2023 to the third lowest severity to date. The median severity in 2023 was 12 days (IQR 9 to 24). This means that the halfway mark of the injury severities was 12 days, with 25% of all Time-Loss injuries lasting 9 days or less and 25% lasting 24 days or longer.

When the medical doctors or medical support staff clinically assessed the injured player, they recorded the injury time from the date that the injury occurred as the starting date. Similarly, the return to play date was recorded when the player returned to full participation in team training and availability for match selection. The injury severity was determined from the difference between these two dates.

These data are grouped to align with the latest IOC statement. The severity groupings include *‘1–7 days’, ‘8–28 days’ and ‘>28 days’* [*2*].

**Figure 6:**
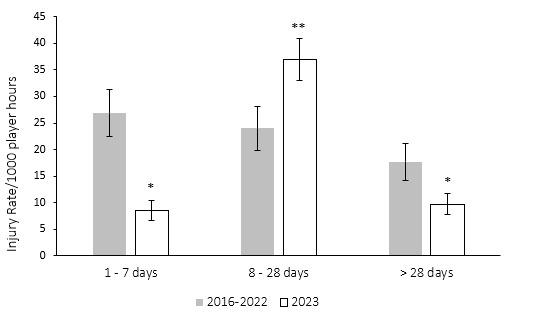
The actual severity category injury rates for the Currie Cup 2023 compared to the averaged injury rates for the actual severity categories for 2016–2022. The whiskers for each bar represent the 95% Confidence Intervals. Asterisk (*) indicates that the injury incidence of 1–7 days and > 28 days lost are significantly lower than their averaged actual severity categories for 2016–2022. Asterisk (**) indicates that the injury incidence of the 8–28 days lost category is significantly higher than its averaged 2016–2022 actual severity.

Figure 6 compares the injury severity rates for the Currie Cup 2023 tournament with the averaged injury severity rates from the 2016–2022 tournaments. Injury rates in the severity category of ‘*1–7 days’* and *‘> 28 days’* were significantly lower in 2023 compared to their 2016–2022 average (Figure 6). Additionally, injury rates in the severity category of ‘*8–28 days’* were significantly higher in 2023 compared to their 2016–2022 average.

Table 2 describes the relationships between incidence, actual severity, and injury burden of each teams’ Time-Loss injuries for the Currie Cup 2023. The Toyota Free State Cheetahs have been used as a worked example to explain Table 2. The Toyota Free State Cheetahs sustained 1.9 injuries per match, meaning that for every 0.5 matches played, they sustained one injury. The Toyota Free State Cheetahs lost 547 training and match days due to injury. This equates to an average of 18 training and match days lost for every injury sustained. The burden of the team’s injuries equates to 1710 days lost per 1000 player hours. Translating this to an operational burden per match shows that the Toyota Free State Cheetahs, within their player group, effectively lost 34.2 days due to injury per match played over the season. The median injury severity for the Toyota Free State Cheetahs was 10 days (IQR 9 to 25). This means that when severities of the Toyota Free State Cheetahs Time-Loss injuries were rank ordered, the midpoint of the severities was 10 days off from rugby, with 25% of their injuries lasting equal to or less than 9 days off, and 25% of their injuries lasting equal to or longer than 25 days off.

**Figure 7:**
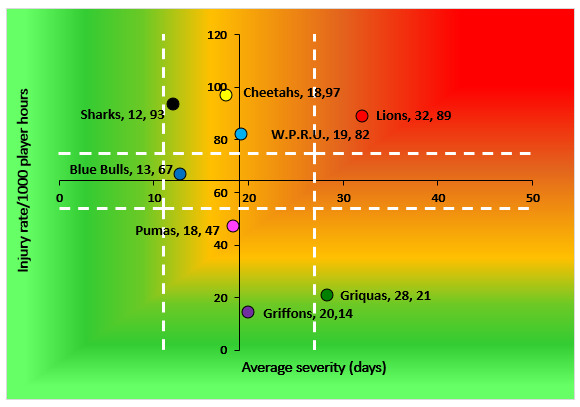
Injury rate plotted against the average severity of Time-Loss injuries for each participating team in the Currie Cup 2023. The Y-axis Average Injury Rate is expressed as the tournament average (the vertical white dotted lines represent 95% Confidence Intervals) and X-axis Average Severity is expressed as the average of all the individual injury severities in the tournament (the horizontal white dotted lines represent 95% Confidence Intervals).

The Toyota Free State Cheetahs had the highest Time-Loss injury rate, followed by the HOLLYWOODbets Sharks and Fidelity ADT Lions. In contrast, the Novavit Griffons had the lowest injury rate, low severity, and, by extension, the lowest injury burden per team (Table 2; Figure 7). Previous studies have shown that teams with lower injury rates succeeded more in the Currie Cup competition [6, 7]. However, this was not the case in the 2023 Currie Cup. It has also been shown that injury burden needs to be considered for success and not simply injury rates alone [8]. Teams who fall in the green zone (below average and 95%CI) will generally not be impacted as much by their injury burden, regardless of whether their injury rate or average severity is relatively high. When the combination of rate and severity moves into the orange (close to average) and/or red zone (above average and 95% CI), the impact on team performance and player availability becomes more problematic. In the 2023 Currie Cup the Fidelity ADT Lions was the only team approaching the red zone. Fidelity ADT Lions also showed the highest injury burden because they combined high injury rates and severity.

All the data in this report are aligned with the 2019 IOC consensus statement [2] and are further presented as such to compare against previous season reports and the international meta-analysis [1]. Table 3 presents the Currie Cup 2023 injury data in the format recommended by the 2019 IOC consensus statement. This table provides an overview of the Tissue and Pathology types of injuries sustained during the 2023 season. This format is used throughout this report.

### New, Subsequent and Recurrent Injuries

During the Currie Cup 2023, the overall injury incidence for *New injuries* was 64 (53 to 74) per 1000 player hours, similar to the injury rate during the Currie Cup 2022.

**Figure 8:**
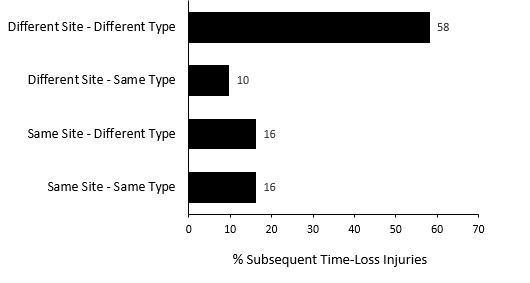
Classification of subsequent injuries for the Currie Cup 2023. Data expressed as a % of subsequent Time-Loss injuries.

Ninety players experienced only one injury during the Currie Cup 2023 season (76% of all injured players). Fifty-eight percent (58%) of subsequent injuries in the 29 players who sustained multiple injury events during the season (Figure 1a and Figure 8), occurred at a different anatomical site and were of a different type compared to the initial index injury. ‘*Different site – different type*’, ‘*different site – same type*’ and ‘*same site – different type*’ are classified as subsequent new injuries. Figure 8 shows the percentage breakdown of subsequent Time-Loss injuries into these categories.

**Figure:**
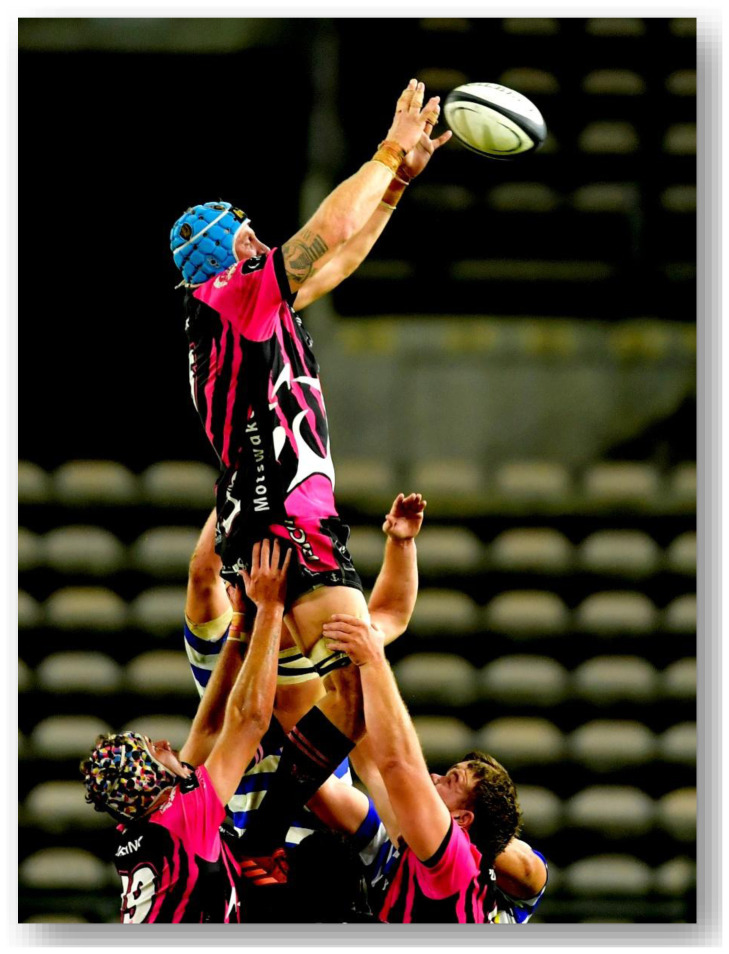


A subsequent *recurrent* injury was any subsequent injury classified as ‘*same site – same type*’, which refers to the same location and same tissue type involved as the original index injury. Only two subsequent recurrent injuries occurred in the Currie Cup 2023.

Subsequent recurrent injury incidence in 2023 was 1 (0 to 2) injury per 1000 player hours, lower than the 2022 tournament’s injury incidence of 3 (0 to 5) injuries per 1000 player hours. However, due to the small number of these injuries, the incidence was not significantly different.

The proportion of new injuries increased, and subsequent recurrent injuries decreased compared to the Currie Cup 2022 tournament (Table 4).

### Injury Type

Overall, the Central Nervous System injuries were the most common Time-Loss injury recorded during the Currie Cup 2023 (28%), followed by Ligament sprain injuries (22%).

The median severity for Central Nervous System injuries was 12 days, with 25% of injuries resulting in 12 or fewer days absent from training and matches and 25% of injuries resulting in 16 or more days absent from training and matches (Table 5). The average severity was 16 days absent.

**Figure 9:**
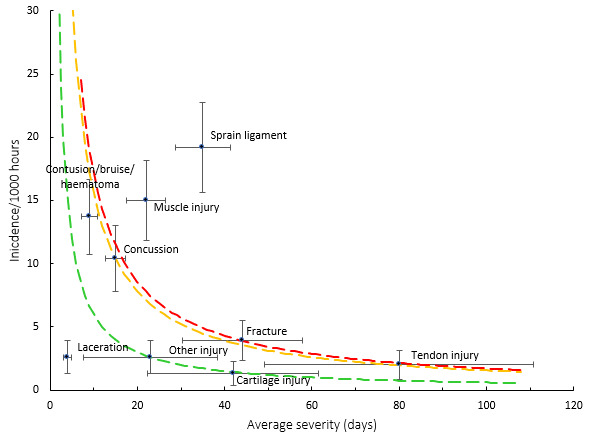
Injury burden as a function of injury type for the seasons 2016 – 2023. The y-axis represents incidence (number of injuries per 1000 hours), and the x-axis represents the average severity (days absent) per injury type. Green line: values to the left and below represent those under the 25^th^ burden percentile; these are low-risk injuries. Orange line: values to the left and below represent those under the 50^th^ burden percentile; these include the low-medium risk injuries. Red line: values to the left and below represent those under the 75^th^ burden percentile; these include the medium-high risk injuries. Values to the right and above the red line are the most high-risk types of injuries and impact players and teams the most.

Figure 9 shows the injury burden for the period 2016–2023. Ligament sprain followed by muscle injury were the two injury types with the highest burden when data were combined for the 2016–2023 Currie Cup tournaments. These injury types have the highest combination of injury incidence and average injury severity. Consistent with previous reports, these two injury types continue to dominate across the different teams.

The most common Time-Loss injuries during the Currie Cup 2023 tournament were central nervous system injuries, recorded at 18 (13 to 24) injuries per 1000 player hours. The average severity for central nervous system injuries was 16 (12 to 19) days.

Following central nervous system injuries, joint (non-bone)/ligament injuries (comprised of dislocation/subluxation and sprain/ligament injuries) were the next most common injuries. Joint (non-bone)/ligament injuries had an injury incidence of 17 (11 to 22) injuries per 1000 player hours. The average severity of joint (non-bone)/ligament injuries in the Currie Cup 2023 was 30 (22 to 37) days. The injury rate for muscle/tendon injuries was 10 (6 to 14) injuries per 1000 player hours. The average severity for muscle/tendon injuries was 24 (9 to 39) days.

### Injury Diagnosis [9]

The most common Orchard Sports Injury Classification System (OSIICS) diagnosis ^[10]^ in the Currie Cup 2023 was Concussion (OSIICS code = HNCX) followed by Hamstring Strain (TMHX) (Table 6).

### Concussions

**Figure 10:**
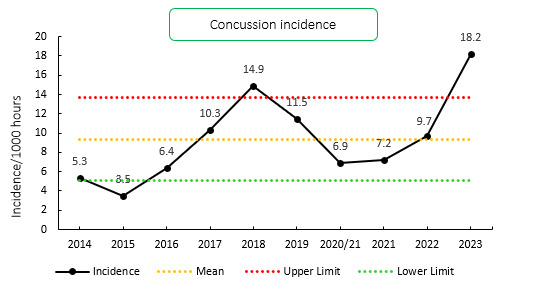
Incidence of concussion over the surveillance period with mean ± standard deviations shown. The red dotted line represents the mean plus standard deviation. The green dotted line represents the mean minus standard deviation. 2020/21 – was a hybrid tournament structure that started in 2020 and carried over into the beginning of the 2021 season due to COVID-19 lockdown interruptions.

Concussions contributed to 43 injuries in the Currie Cup 2023 (28%). Concussion incidence increased from 9.7 injuries per 1000 player exposure hours in 2022 to 18.2 injuries per 1000 player hours in the Currie Cup 2023 tournament. In practical terms, this equates to 0.7 concussions per match, one concussion every 1.4 matches, or one can expect 7 concussions for every 10 matches played. This increase falls outside the expected season-to-season variation for the Currie Cup (Figure 10), with an overall grouped tournament average of 9.4 concussions per 1000 player hours over the entire data collection period. The average severity of concussions reported in the 2023 tournament was 16 days, with a median severity of 12 days (IQR 12 – 16 days). World Rugby approved the use of the Head Injury Assessment (HIA) protocol for the Currie Cup Tournament. Players’ concussion management and Return to Play is per the HIA protocol.

Advanced care clinical settings are defined in the World Rugby and SARU’s Concussion Guideline documents:

- World Rugby HIA protocol: https://www.world.rugby/the-game/player-welfare/medical/concussion/hia-protocol
- World Rugby Concussion Guideline documents: https://www.world.rugby/the-game/player-welfare
- SARU’s Concussion Guideline documents (*When can a player safely return-to-play following a concussion*): www.boksmart.com/concussion, and on *MyBokSmart*: https://my.boksmart.com/Documents/BokSmart#ConcussionManagement

**Figure 11:**
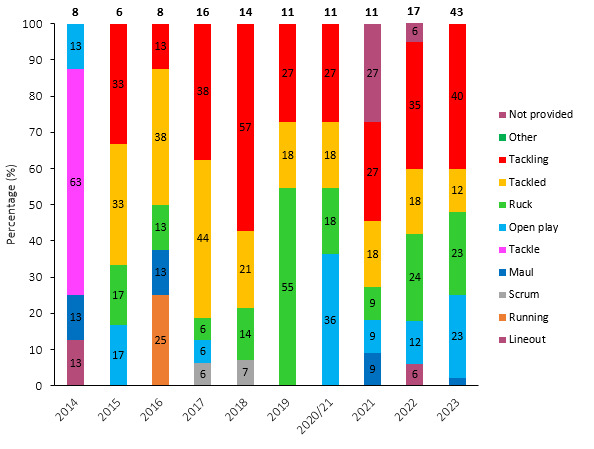
Proportion of concussions caused by the different injury events and total number of concussions from 2014 to 2023. (The number above each bar represents the total number of concussions for that year). 2020/21 – was a hybrid tournament structure that started in 2020 and carried over into the beginning of the 2021 season due to COVID-19 lockdown interruptions.

Figure 11 shows the total number of concussions per year and the proportion of concussions caused by different injury events. The total number of concussions increased dramatically in 2023. The main causes of concussion during the Currie Cup 2023 were *Tackling* (40%), followed by the *Ruck* and *Open Play* (both 23%).

**Figure 12:**
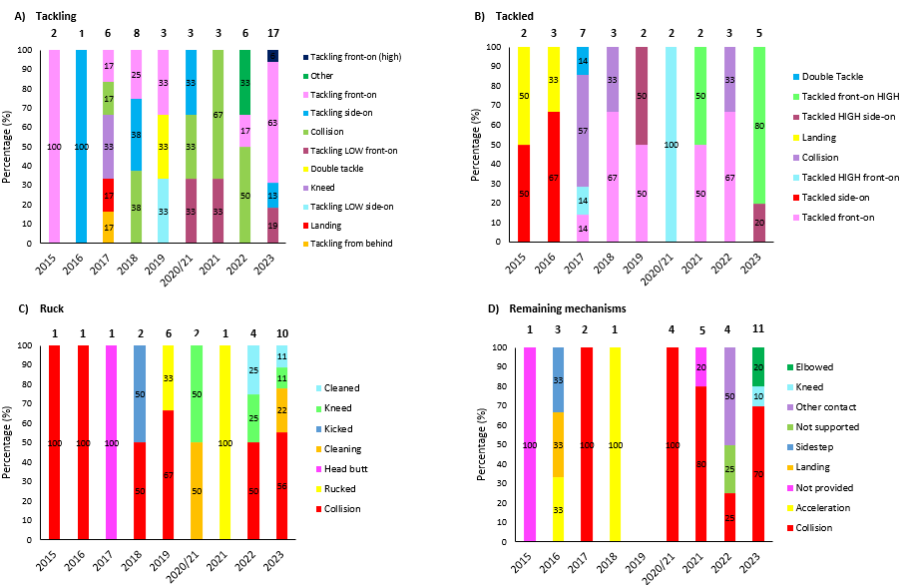
Proportion of concussions caused by A) Tackling, B) Tackled, C) Ruck, and D) Remaining concussion mechanisms from 2015 to 2023. The number above each bar represents the total number of concussions for that event for that year. 2020/21 – was a hybrid tournament structure that started in 2020 and carried over into the beginning of the 2021 season due to COVID-19 lockdown interruptions. Missing cases = 3.

Figure 12 presents the mechanisms contributing to concussions in *Tackling, Tackled, Ruck* and the remaining concussion causing injury events from 2015 – 2023. Data were only presented from 2015 onwards as *Tackle* related data were not captured separately for the *Tackler* and *Ball Carrier* in 2014. *Tackling front on (regulation)* dominated Tackler concussions in 2023 (63%), being *Tackled front-on high* (80%) in ball carriers, *Collisions* in the Ruck (56%), and *Collisions* in Open Play (70%). It was also worth noting that all Tackling-, Ruck-, and the Remaining mechanism-related concussion numbers increased quite noticeably in 2023.

### Injury location

The head was the most frequently injured body location during the Currie Cup 2023 tournament (30%), followed by the knee (20%). Concussions (n = 43) contributed to the most head injuries. Ligament injuries (n = 17) contributed to the most knee injuries. Ligament injuries also accounted for the most ankle injuries (n = 9) and shoulder injuries (n = 4), whereas muscle strain/spasm injuries (n = 13) contributed to the most thigh injuries.

Head injuries had an average severity of 15 days absent and an injury burden of 300 days lost per 1000 player hours. The median severity of head injuries in the Currie Cup 2023 was 12 (IQR 11 to 16) days absent. Twenty-five percent of head injuries resulted in 11 or fewer days lost from training and matches, and 25% of all head injuries resulted in 16 or more days lost from training and matches (Table 7). The average severity for knee injuries was 34 days absent, and the injury burden was 442 days lost per 1000 player hours: the highest injury severity and burden. This was followed by shoulder injuries, with 25 days absent and 175 days lost, respectively. Thigh injuries had the second lowest average severity of 20 days absent and an injury burden of 120 days lost per 1000 player hours.

When analysing the changes in the incidence of the most injured body locations for the Currie Cup over the past eight seasons (2016–2023), the head consistently ranks in the top two on the list. However, the knee, which frequently sits in the top two as well, has increased back to the second most injured body location in 2023, with shoulder injuries having moved down (Table 8).

**Figure:**
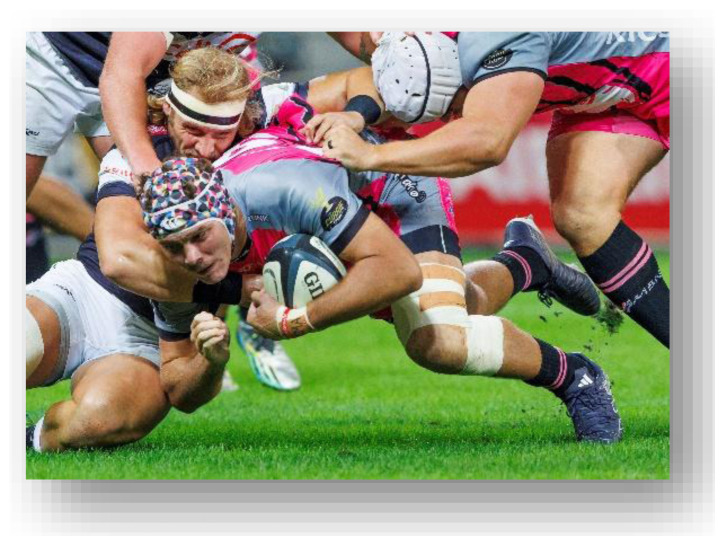


**Figure 13:**
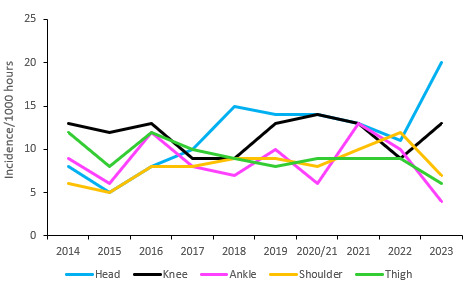
Incidence of the most common injury locations over the surveillance period. 2020/21 – was a hybrid tournament structure that started in 2020 and carried over into the beginning of the 2021 season due to Covid-19 lockdown interruptions.

Figure 13 displays the movement of the most common injured body locations over the surveillance period (2014–2023). When examining the injury incidence patterns over the past ten years, a clear downward trend can be observed in ankle injuries since the 2021 season, after a sharp increase recorded in 2020/21. Shoulder injuries were increasing gradually between 2015 and 2022 but lowered again in 2023. Head injuries reached the highest level recorded in the past nine years. The trend is not the same, but the head injury data here links directly to the concussion section earlier in the report, since most head injuries were attributed to concussions. Thigh injuries had levelled out over the previous three years and then decreased in 2023 (Figure 13).

**Figure 14:**
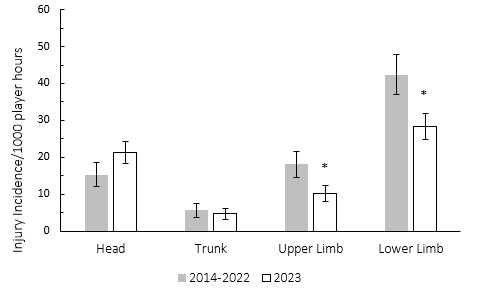
Injury incidence by grouped body location for the Currie Cup 2023 compared to the averaged 2014–2022 injury rates. The whiskers for each bar represent the 95% Confidence Intervals. Asterisk (*) indicates that the injury incidence of 2023’s Upper- and Lower Limb injuries are significantly lower than the averaged 2014–2022 Upper- and Lower Limb injuries.

During the Currie Cup 2023, lower and upper limb injury rates were significantly lower than their 2014–2022 average injury rates (Figure 14). During the Currie Cup 2023, the head and knee areas had the highest injury rates, with 20 (14 to 25) and 13 (8 to 17) injuries per 1000 player hours, respectively. The head injury rate was higher than that of the international meta-analysis [1] of 17 (14 to 20) injuries per 1000 player hours but not significantly. The knee injury rate is similar to the meta-analysis [1] injury rate of 13 (12 to 14) injuries per 1000 player hours.

**Figure:**
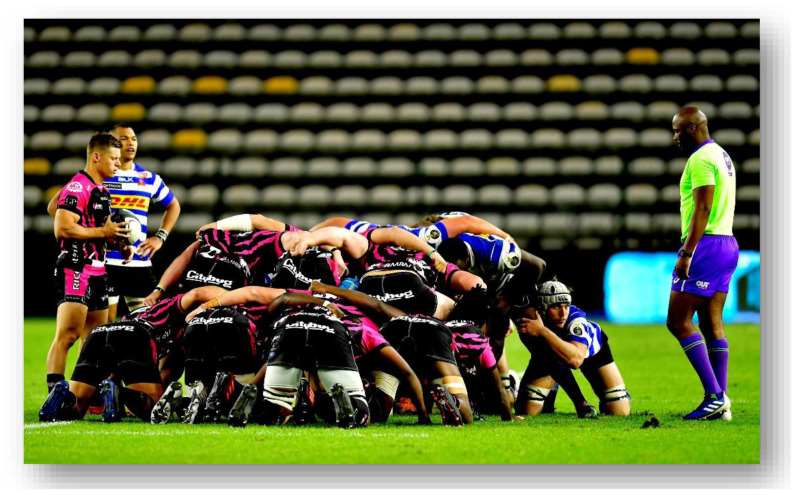


### Injury Event

The *Open Play* event accounted for the most injuries in the Currie Cup 2023 (32%, n = 48), followed by *Tackle (Tackler)*, accounting for 22% of injuries (n = 34) (Table 9). When comparing injury rates to the international meta-analysis, *tackling* at 14 (10 to 19) injuries per 1000 player hours during the Currie Cup 2023 was significantly lower than the meta-analysis results of 23 (21 to 25) injuries per 1000 player hours. *Being tackled* in the Currie Cup 2023 at 13 (9 to 18) injuries per 1000 player hours was also significantly lower than the meta-analysis rate of 23 (21 to 25) injuries per 1000 player hours.

Although lower than the *tackling* incidence in this report, the total severity, average severity, median severity, and burden of injuries to *the ball carrier* were noticeably greater. *Ruck* injury rate during the 2023 season at 8 (4 to 12) injuries per 1000 player hours was similar to the meta-analysis injury rate of 9 (7 to 11) injuries per 1000 player hours [1]. There is no *Open Play* variable in the meta-analysis; therefore, no comparison was made.

**Figure:**
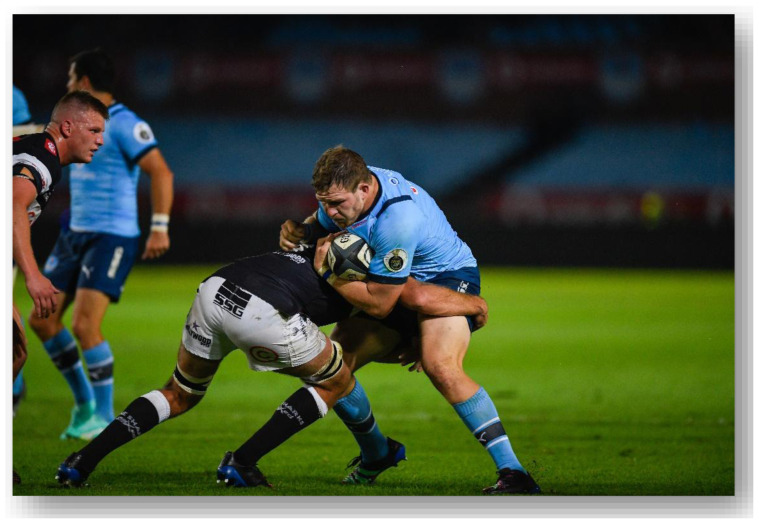


**Figure 15:**
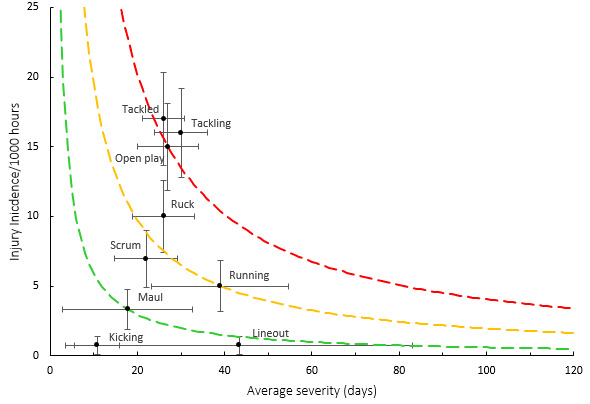
Injury burden as a function of injury event for the seasons 2016 – 2023. The y-axis represents incidence (injuries per 1000 player hours), and the x-axis represents average severity (days absent). Green line: values to the left and below represent those under the 25th burden percentile; these are low-risk injuries. Orange line: values to the left and below represent those under the 50th burden percentile; these include the low-medium risk injuries. Red line: values to the left and below represent those under the 75th burden percentile; these include medium-high risk injuries. Values to the right and above the red line are the most high-risk types of injuries and impact players and teams the most.

Figure 15 combines all the injury events from 2016 – 2023 and presents the injury burden picture over the past eight years. Injuries caused by *Tackling* have the highest injury burden for all teams, followed closely by injuries from *being tackled*. Both these injury events have a high combined injury incidence and average severity. *Open play* followed closely behind these two leading injury-causing events.

**Figure 16:**
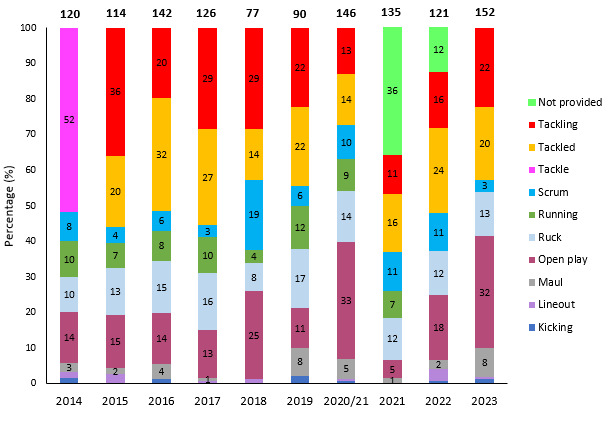
Proportion of injuries caused by the different injury events from 2014 to 2023. (The number above each bar represents that year’s total number of injuries. Tackle data captured separately as tackling and tackled from 2015 onwards). 2020/21 – was a hybrid tournament structure that started in 2020 and continued into the beginning of the 2021 season due to COVID-19 lockdown interruptions.

**Figure 17:**
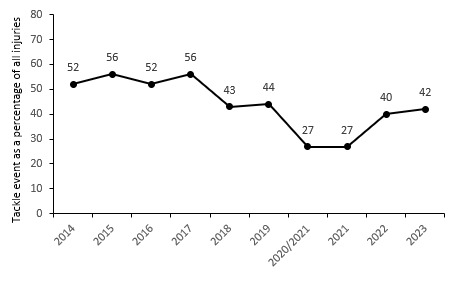
Percentage of all injuries caused by the tackle event from 2014 to 2023. Tackle data were captured separately as tackling and tackled from 2015 onwards. 2020/21 – was a hybrid tournament structure that started in 2020 and continued into the beginning of the 2021 season due to COVID-19 lockdown interruptions.

Figure 16 illustrates the proportion of injuries caused by different events from 2014 to 2023. After a drop in 2018, the rate of injuries caused by tackles has varied over the past six seasons, although it has consistently remained lower than the levels from 2014 to 2017. After a period of decline between 2018 and 2021, the Tackler role has started to rise again, and one needs to monitor this going forward (Figure 17). The tackle event in the last 10 years has contributed on average to 44% of all injury events in the Currie Cup, and since the roles were split into Tacklers and Ball Carriers in 2015, 51% of these have been to Tacklers, and 49% to Ball Carriers.

**Figure 18:**
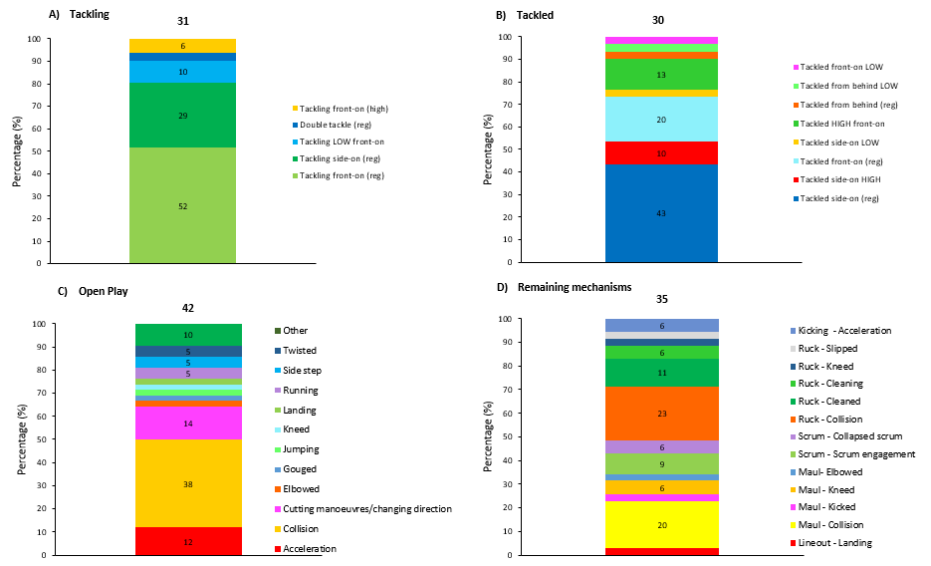
Proportion of injuries per rugby event, caused by A) Tackling, B) Being Tackled, C) during Open Play, and D) the Remaining mechanisms for all other injury causing events in 2023. The number above each bar represents the total number of injuries related to that rugby event for that year. Missing cases = 14.

Figure 18 presents the mechanisms contributing to injuries in *Tackling, Being Tackled, Open Play* and the *Remaining mechanisms* for all other injury-causing events in 2023. *Tackling front-on (regulation)* dominated Tackler injuries in 2023 (52%), *being Tackled side on (regulation)* in ball carriers (43%), *Collisions* in Open Play (38%), and *Collisions* in the Ruck (23%).

**Figure:**
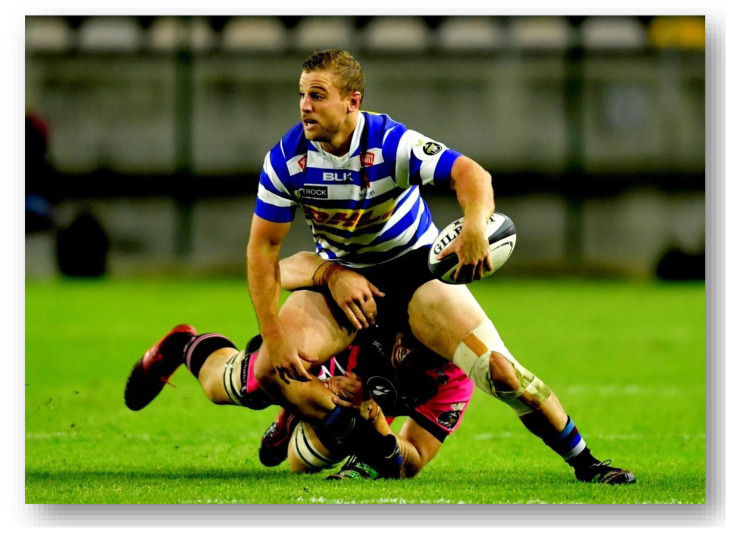


### Venue

**Figure 19:**
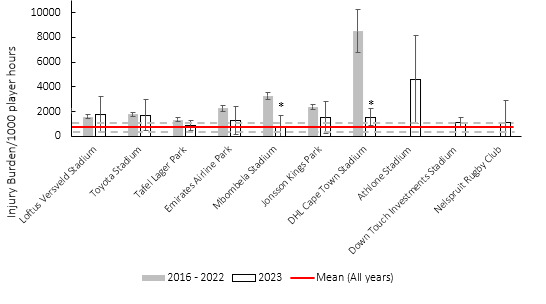
Injury burden/1000 player hours of Time-Loss injuries at the ten utilised stadia in the Currie Cup 2023 in comparison to their averaged 2016–2022 injury burden. *Stadium injury burden was significantly lower in 2023 than 2016–2022 average. The whiskers for each bar represent the 95% confidence intervals.

Matches were played at ten different stadia during the tournament. This is the first year that the Athlone Stadium, Down Touch Investments Stadium and Nelspruit Rugby Club were used during the Currie Cup tournament. Athlone Stadium's injury burden is above the average injury burden (Figure 19), but given the wide confidence intervals, little can be inferred from this at present. In 2023, Mbombela Stadium and DHL Cape Town Stadium's injury burden was significantly lower than their 2016–2022 average.

Table 10 shows the ranking of the injury burden of the Stadia from the highest to lowest between 2016 and 2023. When combining the last eight season’s data, it highlights that the DHL Cape Town Stadium, followed by Athlone Stadium, recorded the highest injury burdens overall, with DHL Cape Town Stadium, Athlone Stadium, and Mbombela Stadium averaged injury burden being significantly higher than the grouped average injury burden from 2016–2023 (Table 10).

**Figure 20:**
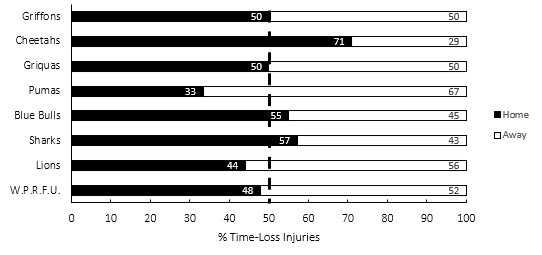
Proportion of injuries sustained playing at home and away venues for the Currie Cup 2023.

Figure 20 presents the proportion of injuries sustained playing at home and away venues in the Currie Cup 2023. When comparing injuries while playing away and at home in the Currie Cup 2023 tournament, playing at home at 31 (23 to 37) injuries per 1000 player hours recorded a similar injury rate to playing away with 34 (27 to 42) injuries per 1000 player hours. The Toyota Free State Cheetahs, Vodacom Blue Bulls and HOLLYWOODbets Sharks, all experienced more injuries when playing at home compared to playing away. In contrast, Airlink Pumas, Fidelity ADT Lions and DHL Western Province Rugby Football Union (W.P.R.F.U) experienced more injuries while playing away compared to at home. Novavit Griffons and Windhoek Draught Griquas had an equal distribution of injuries at home and away matches.

## TRAINING INJURIES

**Figure 21:**
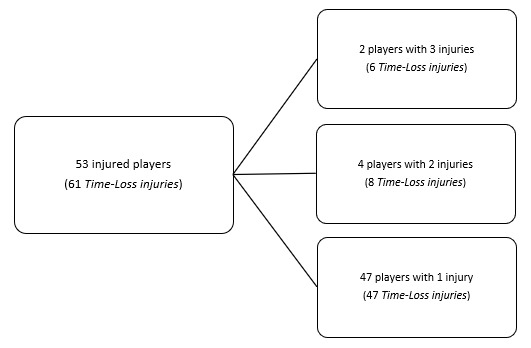
The number of players who experienced Time-Loss injuries in training during the Currie Cup 2023.

**Figure 22:**
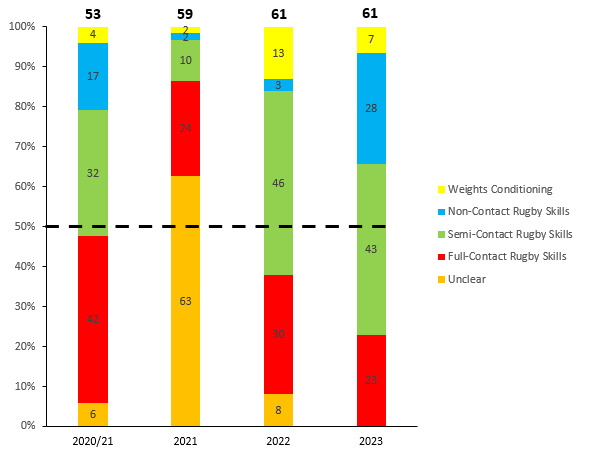
Proportion of Time-Loss training injuries sustained per training activity during the Currie Cup 2020/21-2023. 2020/21 – was a hybrid tournament structure that started in 2020 and continued into the beginning of the 2021 season due to COVID-19 lockdown interruptions.

Overall, 61 Time-Loss injuries were sustained by 53 players during training in the Currie Cup 2023 (Figure 21). The Time-Loss injuries resulted in an injury incidence of 1.5 (1.1 to 1.8) injuries per 1000 training hours, which is lower than the meta-analysis injury incidence of 3.0 (1.9 to 4.0) injuries per 1000 training hours [1]. These Time-Loss injuries contributed to 29% of all injuries experienced during the Currie Cup Tournament over the 2023 rugby season (n = 61 training + 152 matches = 213 injuries in total). The average severity of training injury was 17 days, with a median severity (IQR) of 13 (7 to 19) days absent. Figure 22 shows the percentage of training injuries per training activity. Semi-contact rugby skills accounted for the highest percentage of training injuries, which can be expected given the nature of contact and the time spent involved in those activities. Injuries associated with non-contact training have increased, while full-contact injuries and weights conditioning injuries have decreased.

**Figure:**
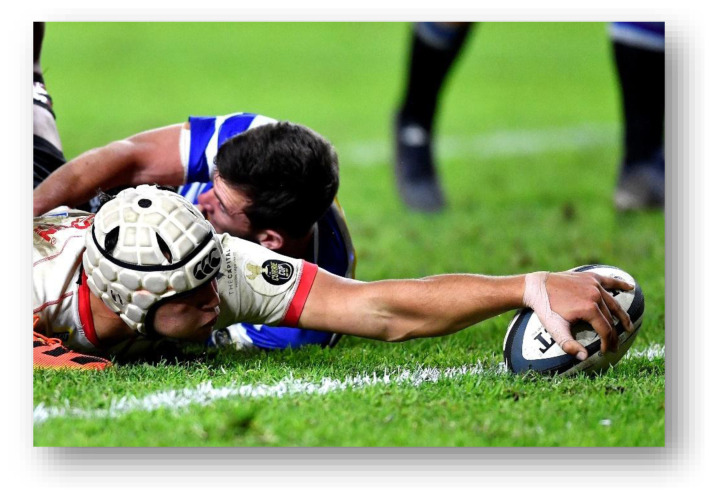


Table 11 presents the training injuries sustained during the Currie Cup 2023. The most common injury type sustained in *full contact* rugby skill activities was shared between *Muscle injury*, *Ligament sprain* and *Joint injury*, with *Joint Injury* having the highest average severity of 45 days lost (Table 11). In *semi-contact* rugby skills, *Muscle injuries* were the most common type, followed by *Ligament sprains*. However, ligament sprains resulted in a greater average severity, with 21 days lost due to injury compared to 11 days for muscle injuries. *Fractures*, although far less common, had the highest average severity of 33 days.

The *thigh* was the most injured body location in training, accounting for 26% (n = 16) of all Time-Loss training injuries during the Currie Cup 2023, followed by the *ankle* (16%) (Table 12). *Wrist/Hand* training injuries clearly had the highest average and median severities, followed by the *Ankle*, and then *Head* training injuries. The *Lower body* as a grouped body location dominated training injuries at 0.9 (0.6 to 1.2) injuries per 1000 player training exposure hours.

**Figure:**
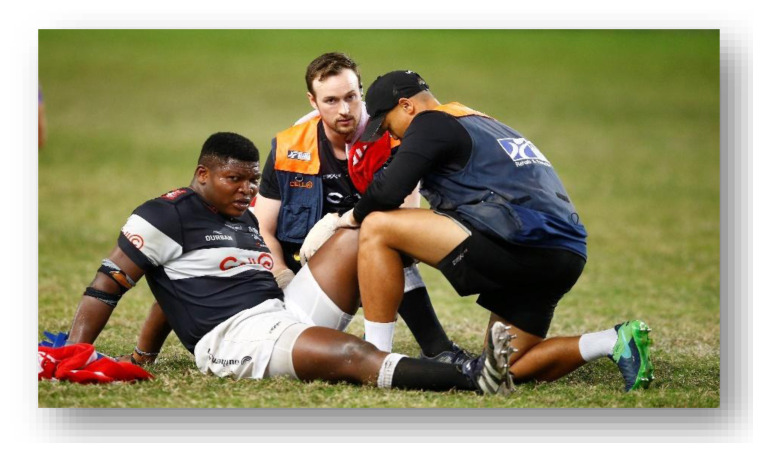


**Figure 23:**
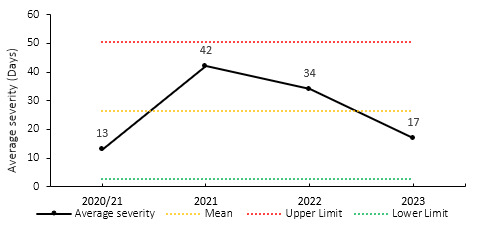
Mean severity of Time-Loss training injuries over the surveillance period with mean ± standard deviations shown. 2020/21 – was a hybrid tournament structure that started in 2020 and continued into the beginning of the 2021 season due to COVID-19 lockdown interruptions.

The average severity of training injuries for the Currie Cup 2023 was 17 days, the second lowest injury severity recorded since the 2020/21 season (13 days) (Figure 23).

**Figure 24:**
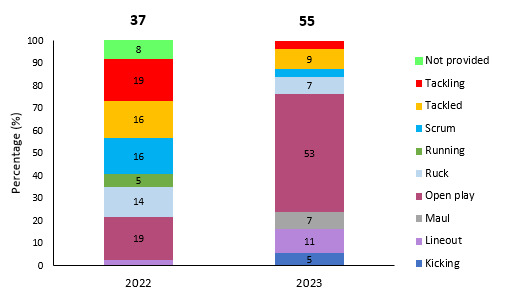
Proportion of injuries caused by the different injury events from 2022 and 2023. (The number above each bar represents the total number of injuries for that year). Missing data in 2022 = 24.

Figure 24 displays the proportion of injuries caused by the different injury events from 2022 and 2023. Strength and conditioning-related injury events were removed due to them not being a rugby-specific event. *Open Play* had the highest proportion of training injuries and increased substantially in 2023 (Figure 24). Injuries in the tackle event (both *Tackling* and *Tackled* player) decreased in 2023.

**Figure 25:**
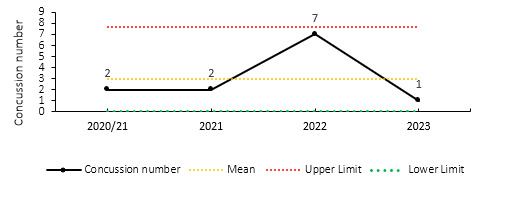
Absolute number of concussions recorded over the training surveillance period. 2020/21 – was a hybrid tournament structure that started in 2020 and carried over into the beginning of the 2021 season due to COVID-19 lockdown interruptions.

Overall, concussions contributed to 1 training injury throughout the Currie Cup 2023 (2%). This is lower than the 2022 Currie Cup season *(*Figure 25).

**Figure 26:**
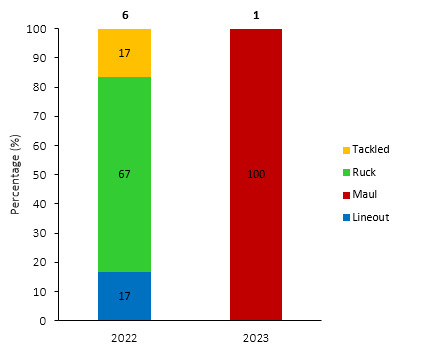
Proportion of concussions caused by the different injury events from 2022 and 2023 during training (The number above each bar represents the total number of concussions for that year). Missing case = 1 case in 2022.

In 2023, the concussion occurred during a collision in the maul, whereas in 2022, most concussions occurred in the ruck *(*Figure 26).

**Figure:**
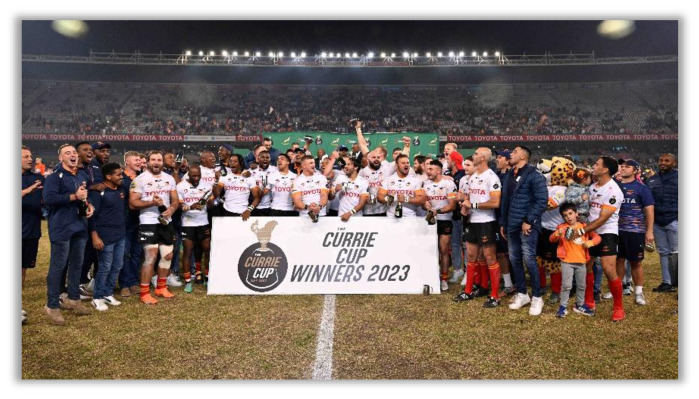


## Figures and Tables

**Figure 1a:**
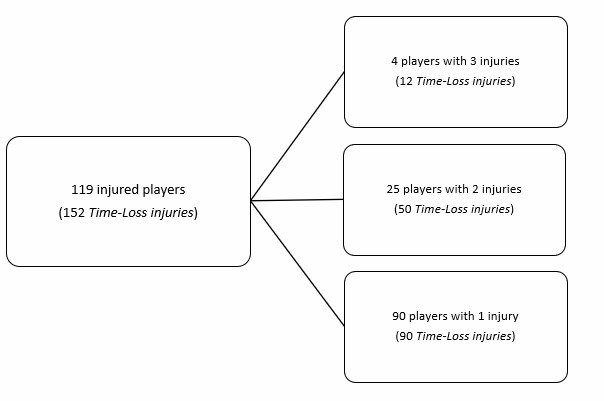
The number of players who experienced Time-Loss injuries during the Currie Cup 2023.

**Figure 1b:**
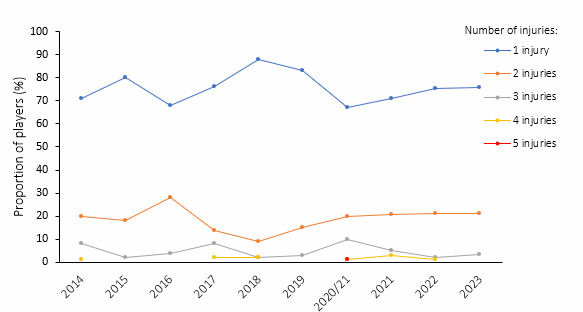
Proportion of injured players experiencing one to five Time-Loss injuries in the Currie Cup tournaments from 2014–2023. 2020/21 – was a hybrid tournament structure that started in 2020 and carried over into the beginning of the 2021 season due to COVID-19 lockdown interruptions.

**Table 1:** Currie Cup tournament formats and competition’s time of year from 2014 to 2023.

Year	Tournament format	Time of year
**2014**	Double round	Second half of the year
**2015**	Double round	Second half of the year
**2016**	Single round	Second half of the year
**2017**	Double round	Second half of the year
**2018**	Single round	Second half of the year
**2019**	Single round	Second half of the year
**2020/21**	Single round	End of year, and beginning of next year
**2021**	Double round	Second half of the year
**2022**	Double round	First half of the year
**2023**	Double round	First half of the year

**Table 2:** Injury Incidence, Severity (days), Injury Burden (days absent/1000 player hours) and Operational Burden (days absent due to injuries/match) of Time-Loss injuries for each participating team in the Currie Cup 2023.

Team	Team Injuries / match	Injury Incidence (per 1000 player hours)	Team matches/ injury	Total Severity(days lost)	Average Severity (days lost/injury)	Injury Burden (days lost / 1000 hours)	Operational Injury Burden (days lost due to injuries /match)	Median Severity (IQR)
Airlink Pumas	0.9	46.9	1.1	276	18	863	17.3	14 (12 to 23)
Vodacom Blue Bulls	1.3	66.7	0.7	256	13	854	17.1	10 (7 to 12)
Toyota Free State Cheetahs	1.9	96.9	0.5	547	18	1710	34.2	10 (9 to 25)
Fidelity ADT Lions	1.8	89.3	0.6	801	32	2861	57.2	23(11 to 44)
HOLLYWOODbets Sharks	1.9	93.3	0.5	337	12	1123	22.5	12 (9 to 20)
DHL Western Province	1.6	82.1	0.6	444	19	1585	31.7	12 (10 to 20)
Windhoek Draught Griquas	0.4	21.4	2.3	170	28	606	12.1	15 (13 to 17)
Novavit Griffons	0.3	14.3	3.5	78	20	279	5.6	19 (18 to 21)
***Overall***	***1.3***	***64.4***	***0.8***	***2909***	***19***	***1232***	***24.6***	***12(9 to 24)***

**Table 3:** The Currie Cup 2023 injuries grouped according to the IOC recommended categories of Tissue and Pathology types for injuries.

Tissue	Incidence	Median time loss	Burden
*Pathology*	Injuries per 1000 hours (95%CI)	Days (95%CI)	*Days per 1000 hours (95%CI)*
**Muscle/Tendon**	**10 (6 to 14)**	**10 (0 to 25)**	**240 (144 to 336)**
Muscle Injury	10 (5 to 13)	10 (2 to 18)	160 (80 to 208)
Tendinopathy	1 (0 to 2)	163	163
**Ligament/Joint capsule**	**17 (11 to 22)**	**23 (15 to 44)**	**510 (330 to 660)**
Ligament Sprain	14 (10 to 19)	23 (12 to 43)	392 (280 to 532)
Joint Sprain	2 (0 to 4)	48 (33 to 63)	96 (0 to 192)
**Nervous**	**20 (14 to 26)**	**12 (9 to 15)**	**300 (210 to 390)**
Brain/Spinal cord injury	18 (13 to 24)	12 (8 to 16)	288 (208 to 384)
Peripheral nerve injury	2 (0 to 3)	13 (0 to 25)	30 (0 to 45)
**Superficial tissues/skin**	**8 (4 to 12)**	**9 (0 to 20)**	**144 (72 to 216)**
Contusion (superficial)	8 (4 to 12)	9 (0 to 20)	144 (72 to 216)
**Bone**	**6 (3 to 10)**	**16 (11 to 57)**	**216 (108 to 360)**
Fracture	3 (1 to 5)	30 (14 to 64)	117 (39 to 1954)
Bone contusion	4 (1 to 6)	14 (2 to 67)	140 (35 to 210)
**Cartilage/Synovium/Bursa**	**2 (0 to 4)**	**21 (3 to 39)**	**50 (0 to 100)**
Bursitis/Synovitis	2 (0 to 4)	**21 (3 to 39)**	50 (0 to 100)
**Non-specific**	**2 (0 to 3)**	16 (4 to 27)	30 (0 to 45)

***Overall***	***64 (54 to 75)***	***12 (8 to 16)***	***1232 (1033 to 1435)***

**Table 4:** Proportion (%) of new versus subsequent recurrent injuries for the Currie Cup 2016 – 2023 tournaments.

	2016	2017	2018	2019	2020/21	2021	2022	2023
New injuries	74	74	86	83	68	71	78	98.7
Subsequent recurrent injuries	2.8	3.2	2.6	2.2	3.4	4.4	3.3	1.3

**Table 5:** Injury rate, Severity and Burden of the most common injury types in the Currie Cup 2023.

Injury Type	Injury Rate (*95%* CI) *(per 1000 hours)*	Total Severity *(days)*	Average Severity *(days)*	Burden (*95%* CI) *(days lost / 1000 hours)*	Median *(IQR)*
Central Nervous System	18 (13 to 24)	639	16	288 (208 to 384)	12 (12 to 16)
Sprain Ligament	14 (10 to 19)	736	28	392 (280 to 532)	23 (12 to 42)
Muscle (Rupture/Strain/Tear)	10 (5 to 13)	312	16	160 (80 to 208)	10 (8 to 18)
Contusion/Bruise	8 (4 to 12)	321	18	144 (72 to 216)	9 (7 to 12)
Broken Bone/Fracture	3 (1 to 5)	194	39	117 (39 to 195)	30 (11 to 72)

***Overall***	***64 (54 to 75)***	***2909***	***19***	***1232 (1033 to 1435)***	***12 (9 to 24)***

**Table 6:** The movement of the most common OSIICS classification diagnoses over the past eight seasons [9]. 2020/21 – was a hybrid tournament structure that started in 2020 and continued into the beginning of the 2021 season due to COVID-19 lockdown interruptions.

			Percentage (%)	Number	Incidence (*95%* CI)	Average Severity
2016		Concussion (HN1)	7	10	6 (2–10)	14
		Knee medial collateral ligament strain/tear/rupture (KL3)	6	9	6 (2–10)	23
		Hamstring strain/tear (TM1)	6	8	5 (2–9)	11
2017		Concussion (HNCX)	13	16	10 (5–15)	15
		Acromioclavicular joint sprain (SJAX)	10	12	8 (3–12)	25
2018		Concussion (HNCX)	18	14	15 (7–23)	14
		Quadricep strain (TMQX)	5	4	4 (0–8)	18
2019		Concussion (HNCX)	12	11	12 (5–18)	9
		Ankle syndesmosis sprain (AJSX)	5	5	5 (1–10)	14
2020/21		Concussion (HNCX)	8	11	7 (3–11)	10
		Quadriceps haematoma (THV)	4	6	4 (1–7)	4
		Knee strain (MCL)	3	5	3 (1–6)	42
2021		Concussion (HNCX)	8	11	7 (3–12)	15
		Ankle sprain (AJXX)	6	8	6 (2–10)	10
		Hamstring strain (THHX)	2	5	2 (0–4)	23
2022		Concussion (HNCX)	14	17	10 (5–14)	24
		Hamstring strain (TMHX)	6	7	4 (1–7)	46
		Ankle syndesmosis sprain (AJSX)	4	5	3 (0–5)	12
2023		Concussion (HNCX)	28	43	18 (13–24)	16
		Hamstring strain (TMHX)	5	7	3 (1–5)	17
		Ankle sprain (AJXX)	3	4	2 (0–3)	19
		Grade 1 medial collateral ligament tear knee (KJMA)	3	4	2 (0–3)	11

**Table 7:** Injury rate, Severity and Burden of the most common injured body locations in the Currie Cup 2023.

Injury Location	Injury Rate (*95%* CI) *(per 1000 hours)*	Total Severity *(days)*	Average Severity *(days)*	Burden (*95%* CI) *(days lost / 1000 hours)*	Median *(IQR)*
Head	20 (14 to 25)	665	15	300 (210 to 375)	12 (11 to 16)
Knee	13 (8 to 17)	776	34	442 (272 to 578)	25 (10 to 48)
Shoulder	7 (4 to 11)	328	25	175 (100 to 275)	16 (7 to 24)
Thigh	6 (3 to 9)	238	20	120 (60 to 180)	11 (8 to 27)
Ankle	4 (2 to 7)	223	25	100 (125 to 175)	19 (18 to 37)

***Overall***	***64 (54 to 75)***	***2909***	***19***	***1232 (1033 to 1435)***	***12 (9 to 24)***

**Table 8:** Movement of the most injured body locations over the past eight seasons. 2020/21 – was a hybrid tournament structure that started in 2020 and carried over into the beginning of the 2021 season due to COVID-19 lockdown interruptions.

			Percentage (%)	Number	Incidence (95% CI)	Average Severity
2016		Knee	14	20	13 (7–18)	49
		Ankle	13	18	12 (6–17)	51
		Head	9	13	8 (4–13)	11
		Shoulder	8	12	8 (3–12)	41
2017		Head	13	16	10 (5–15)	15
		Knee	11	14	9 (4–14)	63
		Shoulder	10	12	8 (3–12)	67
		Ankle	10	12	8 (3–12)	87
		A/C Joint	10	12	8 (3–12)	25
2018		Head	18	14	15 (7–23)	18
		Knee	10	8	9 (3–14)	44
		Shoulder	10	8	9 (3–14)	38
		Ankle	9	7	7 (2–13)	65
		Anterior thigh	8	6	6 (1–12)	6
2019		Head	14	13	14 (6 – 21)	8
		Knee	13	12	13 (5 – 20)	13
		Ankle	11	10	10 (4 – 17)	9
		Lower limb posterior	7	6	6 (1 – 11)	3
		Posterior thigh	7	6	6 (1 – 11)	9
2020/21		Head	16	23	14 (9 to 20)	6
		Knee	15	22	14 (8 to 19)	57
		Thigh	10	15	9 (5 to 14)	9
		Shoulder	9	13	8 (4 to 13)	22
		Ankle	7	10	6 (2 to 10)	19
2021		Head	15	20	13 (7 to 19)	9
		Knee	15	20	13 (7 to 19)	41
		Ankle	14	19	13 (7 to 18)	13
		Shoulder	11	15	10 (5 to 15)	18
		Thigh	10	14	9 (4 to 14)	22
2022		Shoulder	17	21	12 (7 to 17)	55
		Head	17	20	11 (6 to 16)	24
		Ankle	15	18	10 (5 to 15)	30
		Knee	13	16	9 (5 to 14)	46
		Thigh	13	16	9 (5 to 14)	29
2023		Head	30	46	20 (14 to 25)	15
		Knee	20	30	13 (8 to 17)	34
		Shoulder	11	17	7 (4 to 11)	25
		Thigh	9	14	6 (3 to 9)	20
		Ankle	7	10	4 (2 to 7)	25

**Table 9:** Injury rate, Severity and Burden of the injury events in the Currie Cup 2023

*Injury event*	Injury Rate (*95%* CI) *(per 1000 hours)*	Total Severity *(days)*	Average Severity *(days)*	Burden (*95%* CI) *(days lost / 1000 hours)*	Median*(IQR)*
Open play	20 (15 to 26)	934	19	380 (285 to 494)	12 (9 to 23)
Tackle (Tackler)	14 (10 to 19)	590	17	238 (170 to 323)	12 (11 to 24)
Tackle (Ball Carrier)	13 (9 to 18)	720	23	299 (207 to 414)	14 (9 to 26)
Ruck	8 (4 to 12)	442	23	184 (92 to 276)	14 (11 to 24)
Maul	5 (2 to 8)	129	11	55 (22 to 88)	10 (7 to 15)
Scrum	2 (0 to 4)	50	10	20 (0 to 40)	16 (13 to 20)
Kicking	1 (0 to 2)	25	13	10 (0 to 20)	10 (9 to 10)
Lineout	0.4 (0 to 1)	25	25	10 (0 to 25)	25
***Overall***	***64 (54 to 75)***	***2909***	***19***	***1232 (1033 to 1435)***	***12 (9 to 24)***

**Table 10:** Injury burden/1000 hours of Time-Loss injuries at the ten Stadia utilised in the Currie Cup combined data from 2016 to 2023.

*Stadium*	*Burden (95%Cl)*
**DHL Cape Town Stadium**	**4938 (4065 to 5810)**
**Athlone Stadium**	**4601 (2974 to 6228)**
**Mbombela Stadium**	**2931 (2667 to 3194)**
Jonsson Kings Park	2411 (2221 to 2600)
Emirates Airline Park	2077 (1888 to 2265)
Toyota Stadium	1763 (1621 to 1904)
Loftus Versveld Stadium	1615 (1477 to 1753)
Tafel Larger Park	1262 (1133 to 1392)
Nelspruit Rugby Club	1100 (322 to 1878)
Down Touch Investments Stadium	1086 (743 to 1429)
***Grouped Average***	***2081 (1891 to 2271)***

**Table 11:** Injury incidence rate, average-, and median severity of training injuries sustained during the Currie Cup 2023 season according to the type of training activity involved.

	Injury Incidence (per 1000 playerhours)	Average severity(days)	Median severity(days)
***Rugby skills (full contact)***	***0.3 (0.2 to 0.5)***	***25***	***17***
** ** **Muscle Injury**	0.1 (0.0 to 0.2)	29	8
** ** **Ligament Sprain**	0.1 (0.0 to 0.2)	18	16
** ** **Joint injury**	0.1 (0.0 to 0.2)	45	45
** ** **Nerve Injury**	0.0 (0.0 to 0.1)	14	14
***Rugby skills (semi-contact)***	***0.6 (0.4 to 0.9)***	***14***	***12***
** ** **Muscle Injury**	0.3 (0.1 to 0.4)	11	9
** ** **Ligament Sprain**	0.2 (0.1 to 0.3)	21	21
** ** **Bruising/Haematoma**	0.0 (0.0 to 0.1)	8	8
** ** **Unspecified**	0.0 (0.0 to 0.1)	6	6
** ** **Concussion**	0.0 (0.0 to 0.1)	12	12
** ** **Fracture**	0.0 (0.0 to 0.1)	33	33
** ** **Tendon Injury**	0.0 (0.0 to 0.1)	15	15
***Rugby skills (non-contact)***	***0.4 (0.2 to 0.6)***	***18***	***13***
** ** **Muscle Injury**	0.3 (0.1 to 0.4)	11	12
** ** **Ligament Sprain**	0.1 (0.0 to 0.2)	43	43
** ** **Unspecified**	0.0 (0.0 to 0.1)	32	32
** ** **Bruising/Haematoma**	0.0 (0.0 to 0.1)	7	7
***Weights conditioning***	***0.1 (0.0 to 0.2)***	***12***	***12***
*** *** ***Overall***	***1.5 (1.1 to 1.8)***	***17***	***13***

**Table 12:** Injury incidence rate, average-, and median severity of training injuries sustained per body location, during the Currie Cup 2023.

	Injury Incidence (per 1000 player hours)	Average severity (days)	Median severity (days)
***Head***	***0.0 (0.0 to 0.1)***	***23***	***23***
***Neck***	***0.1 (0.0 to 0.2)***	***8***	***7***
***Upper Body***	***0.2 (0.0 to 0.3)***	***15***	***9***
** ** **Shoulder**	0.1 (0.0 to 0.2)	10	10
** ** **Upper arm**	0.0 (0.0 to 0.1)	9	9
** ** **Wrist/Hand**	0.0 (0.0 to 0.1)	45	45
***Lower Body***	***0.9 (0.6 to 1.2)***	***19***	***12***
** ** **Ankle**	0.2 (0.1 to 0.4)	26	24
** ** **Foot**	0.0 (0.0 to 0.1)	12	12
** ** **Hip/Groin**	0.1 (0.0 to 0.2)	26	9
** ** **Knee**	0.1 (0.0 to 0.2)	15	15
** ** **Lower Leg**	0.1 (0.0 to 0.2)	14	8
** ** **Thigh**	0.4 (0.2 to 0.6)	14	12
***Trunk***	***0.2 (0.1 to 0.3)***	***14***	***15***
** ** **Buttock/Pelvis**	0.0 (0.0 to 0.1)	15	15
** ** **Chest**	0.0 (0.0 to 0.1)	17	17
** ** **Lumbar Spine**	0.1 (0.0 to 0.2)	15	15
** ** **Thoracic Spine**	0.0 (0.0 to 0.1)	5	5
*** *** ***Overall***	***1.5 (1.1 to 1.8)***	***17***	***13***
